# Integrated transcriptomic and metabolomic analysis of Jinggang honey pomelo yellow spot disease to reveal the disease resistance mechanism

**DOI:** 10.1371/journal.pone.0330626

**Published:** 2025-09-04

**Authors:** Huimin Sun, Zexia Li, Yongfa Guo, Weiwei Li, Biling Jin, Ling Lu, Dingkun Liu, Li He, Shuaiwen Yin, Yang Wu

**Affiliations:** College of Life Sciences, Jinggangshan University, Ji’an City, Jiangxi Province, China; Anhui University of Chinese Medicine, CHINA

## Abstract

Jinggangshan honey pomelo is a specialty fruit grown in Jiangxi Province, China. Pomelo yellow spot disease, also known as greasy spot disease, is a fungal pathology primarily affecting pomelo (*Citrus maxima*) leaves and fruits. The causative agent is the ascomycete fungus Phyllosticta citricarpa, taxonomically classified within the phylum Ascomycota.This study performed integrated metabolomic and transcriptomic analysis to identify key differentially accumulated metabolites (DAMs) and differentially expressed genes (DEGs) between the control (CK) and diseased groups (HB) of the Jinlan pomelo (af) and Jinsha pomelo (js) varieties. We identified 1,681 DAMs in the af variety and 1,233 DAMs in the jsCK_vs_jsHB variety. Flavonoid compounds were the most upregulated class of DAMs in both the af and js diseased varieties. Transcriptome analysis identified 1,714 common DEGs between the af and js diseased varieties. KEGG pathway enrichment analysis demonstrated that these common DEGs were significantly enriched in pathways such as plant hormone signal transduction, plant-pathogen interaction, phenylpropanoid biosynthesis, MAPK signaling pathway in plants, and flavonoid biosynthesis. The integrated metabolome and transcriptome analysis showed that the metabolic pathways associated with the common DEGs and DAMs in both the af and js varieties were significantly enriched in phenylpropanoid biosynthesis pathway. Our data showed that differential expression of key enzymes in the flavonoid biosynthesis and phenylpropanoid biosynthesis pathways led to the accumulation of flavonoid metabolites, which play a crucial role in the plant defense mechanisms against external stresses, including pathogen infection. The results suggest that the flavonoid compounds play a key role in the disease resistance mechanism of Jinggangshan honey pomelo against yellow spot disease.

## 1. Introduction

Jinggang honey pomelo, a fruit specialty associated with the “Jinggangshan” brand, belongs to the Aurantinoideae subfamily in the Rutaceae family. Pomelos have been cultivated in China for over 4,000 years and are predominantly produced in regions such as Guangxi, Guangdong, and Chongqing where the climate is hot and humid and is ideal for the growth of pomelos. However, these conditions are also conducive for the proliferation of various pathogens and insects [1]. Pomelo yellow spot disease, also known as greasy spot disease, is a fungal pathology primarily affecting pomelo (*Citrus maxima*) leaves and fruits. The causative agent is the ascomycete fungus Phyllosticta citricarpa, taxonomically classified within the phylum Ascomycota.The pathogen survives the winter as mycelium in diseased and fallen leaves. In the spring, perithecia release ascospores, which are disseminated by wind and rain. These ascospores attach to the young leaves, develop into extracellular mycelium, and produce conidia. The conidia invade branches, leaves, and fruits through the stomata. Subsequently, this pathogen spreads from the infected trees to the adjacent healthy trees. The yellow spot pathogen thrives optimally at temperatures ranging from 25–30°C. Furthermore, significant rainfall is required for disease outbreak. Consequently, excessive rainfall and high humidity contributes to severe disease incidence.

Yellow spot disease in Jinggang honey pomelo damages the branches, leaves, and fruits. The initial symptoms are observed on the leaves and are characterized by the emergence of tiny, translucent, pale green spots on the lower epidermis. These gradually enlarge into yellowish lesions of various sizes. Blister-like, pale yellowish swellings occur in clusters on the abaxial surface of leaves. These lesions are irregularly dispersed across the affected leaves. They are often concentrated on one side of the leaf midrib while the opposite side remains spot-free. Subsequently, the infection leads to substantial defoliation and significantly impacts the vitality of tree and fruit production. In the infected fruits, yellow irregular circular spots appear on the surface and subsequently transform into dark brown spots. These spots are distributed either singly or in clusters, thereby impacting their appearance and commercial value. The management of this disease primarily focuses on prevention. Early spring protective measures and selection of effective curative fungicides are crucial for disease control.

In recent years, rapid advances in omics technologies have facilitated in-depth understanding of the functions, types, and quantitative analysis of plant secondary metabolites and their biosynthetic pathways. Integration of multi-omics technologies provides a powerful approach to understand the complex biological changes in living organisms [2]. Fu et al. [3] performed integrated metabolomic and transcriptomic analyses to identify the pathogenic factors of *Ustilaginoidea virens*. Li et al. [4] used a combination of transcriptomics and metabolomics to elucidate mechanisms that regulate the accumulation of flavonoid metabolites during the development of fruits with diverse colors. Meng et al. [5] used an integrated analysis method of metabolomics and transcriptomics to explore the biosynthetic pathway of flavonoids in *Ginkgo biloba*. This provided significant insights for metabolic engineering research focusing on flavonoid biosynthesis in *Ginkgo biloba*. The complexity of plant responses to external stresses cannot be sufficiently described by a single omics approach.

This study used both metabolomics and transcriptomics to identify differentially accumulated metabolites (DAMs) and differentially expressed genes (DEGs) and analyzed the correlations between them to comprehensively identify defense mechanisms against yellow spot disease in the Jinggang honey pomelo. By establishing a connection between genes and phenotypes, we aimed to elucidate the metabolic pathways of plants from both the “cause” and “effect” standpoints, thereby uncovering the metabolic regulatory network in Jinggang honey pomelo. Based on this, we aimed to identify key molecules involved in the tolerance responses of pomelo to external stresses, thereby providing vital information for future studies on yellow spot disease in Jinggang honey pomelo [3].

## 2. Materials and methods

### 2.1. Sample preparation

Fruits of two varieties exhibiting symptoms of yellow spot disease, Jinlan pomelo (designated as ‘af’) and Jinsha pomelo (designated as ‘js’), were collected from Ji’an City, Jiangxi Province, China. Healthy and disease-affected pomelos were carefully segregated from the Jinggang honey pomelo cultivars and transported to the laboratory for further analysis. In the laboratory, they were rinsed with tap water at arrival and air-dried at ambient temperature. The peels of both healthy and disease-affected pomelo were carefully separated along the equator, while avoiding contact with the interior flesh or the edible portion. The peels were ground into a fine powder in liquid nitrogen and stored at −80°C for subsequent extraction processes.

### 2.2. Metabolomics analysis

#### 2.2.1. Metabolites extraction and machine testing.

We extracted 50 mg of each sample with 1 ml of solvent (methanol:acetonitrile: water; 2:2:1 ratio; v/v/v) that also included 20 mg/L internal standard. The mixture was vortexed for 30 s to ensure thorough mixing. Then, the samples were homogenized with steel beads using a grinding instrument at 45 Hz for 10 min followed by sonication in an ice-water bath for 10 min. The samples were then incubated at −20 °C for 1 h and subsequently centrifuged at 12,000 rpm for 15 min at 4 °C. The resulting supernatant (500 µL) was carefully transferred into an EP tube and dried using a vacuum concentrator.

The dried extract was reconstituted in 160 µL of extraction solvent (acetonitrile:water, 1:1, v/v), vortexed for 30 s, and sonicated again in an ice-water bath for 10 min. The samples were centrifuged again at 12,000 rpm for 15 min at 4 ◦C and 120 µL of the supernatant was transferred into a 2 mL vial for metabolomic analysis. The quality control (QC) sample was prepared by pooling 10 µL of supernatant from each individual sample. The LC/MS system setup for the metabolomics analysis consisted of a Waters Acquity I-Class PLUS ultra-high performance liquid chromatograph coupled with a Waters Xevo G2-XS QTOF high-resolution mass spectrometer and was conducted in a Waters Acquity UPLC HSS T3 column (1.8 µm, 2.1 × 100 mm). For the positive ion mode, we used 0.1% formic acid aqueous solution for mobile phase A and 0.1% formic acid in acetonitrile for mobile phase B. For the negative ion mode, mobile phases A and B were identical to those used in the positive ion mode. The injection volume was set at 1 µL.

#### 2.2.2. LC-MS analysis.

In each data acquisition cycle, dual-channel data collection was performed simultaneously at low and high collision energies. The low collision energy was set at 2 V and the high collision energy ranged from 10 to 40 V. The mass spectrometry scan frequency was 0.2 seconds. The parameters for the ESI ion source were as follows: capillary voltage, 2000 V for positive ion mode and −1500 V for negative ion mode; cone voltage, 30 V; ion source temperature, 150°C; desolvation gas temperature, 500°C; nebulizer gas flow rate, 50 L/h; and desolvation gas flow rate, 800 L/h.

#### 2.2.3. LC/MS data preprocessing and annotation.

Raw data was collected using the MassLynx V4.2 software (Waters Corporation, Shanghai, China) and processed using the Progenesis QI software. This software performed peak extraction, peak alignment, and other data processing operations. Subsequently, the compounds were identified by referencing the online METLIN database (https://ngdc.cncb.ac.cn/databasecommons/database/id/ 5907) and Biomark’s self-constructed library. The theoretical fragment identification and mass deviation was maintained within 100 ppm.

#### 2.2.4. Metabolomic data analysis.

Follow-up analysis was performed after normalizing the original peak area with the total peak area. Both principal component analysis (PCA) and Spearman correlation analysis were performed to assess repeatability of the experimental and quality control samples. The classification and pathway information of the identified compounds was identified using the KEGG, HMDB and LIPID MAPS databases. Based on the grouping information, fold changes were calculated for various compounds between the groups. T test was used to determine if the statistical differences were significant based on the p-values for each compound. The R language package ropls was used to perform orthogonal partial least squares discriminant analysis (OPLS-DA) modeling, and 200 permutation tests were performed to verify the reliability of the model. The variable importance in projection (VIP) value of the model was calculated by multiple cross-validation. Differential metabolites were screened based on the fold change (FC) values, P values, and VIP values of the OPLS-DA model using FC > 1, P value<0.05, and VIP > 1 as threshold parameters. The enriched KEGG pathways based on the differential metabolites were identified using the hypergeometric distribution test.

### 2.3. Transcriptomic analysis

#### 2.3.1. RNA extraction.

Total plant RNA was extracted using the RNAprep Pure Plant Kit (Tiangen, Beijing, China) according to manufacturer’s instructions.

#### 2.3.2. Library preparation for transcriptome sequencing.

Sequencing libraries were constructed with 1 μg RNA samples using the Hieff NGS Ultima Dual-mode mRNA Library Prep Kit for Illumina (Yeasen Biotechnology (Shanghai) Co., Ltd.) according to the manufacturer’s guidelines. Index codes were incorporated to assign sequences to each sample. Firstly, mRNA was purified from total RNA samples using the poly-T oligo-attached magnetic beads. Then, the first strand of cDNA was synthesized followed by synthesis of the second strand. Exonuclease/polymerase activities were used to convert overhangs into blunt ends. The 3’ ends of the DNA fragments were adenylated. NEBNext Adaptors with a hairpin loop structure were ligated to the A-tailed DNA fragments to prepare them for hybridization. The library fragments were then purified using the AMPure XP system (Beckman Coulter, Beverly, USA). The size-selected, adaptor-ligated cDNA was incubated with 3 μl of USER Enzyme (NEB, USA) at 37°C for 15 minutes. Then, after denaturation at 95°C for 5 minutes, PCR amplification was performed using Phusion High-Fidelity DNA polymerase, universal PCR primers, and index (X) Primers. Finally, PCR products were purified using the AMPure XP system, and the library quality was assessed on the Agilent Bioanalyzer 2100 system. The libraries were sequenced according to the manufacturer’s instructions on an Illumina NovaSeq platform to generate 150 bp paired-end reads.

#### 2.3.3. Transcriptome data analysis.

The raw reads were further processed and analyzed using the BMKCloud bioinformatics analysis platform (http://www.biocloud.net). Raw reads in fastq format were initially processed using in-house Perl scripts. During this process, clean reads were filtered by eliminating adaptor sequences, reads containing poly-N, and low-quality reads from the raw data. Then, Q30 score, GC content, and sequence duplication level of the clean reads were calculated. Subsequent analyses were conducted using high-quality clean data.

The clean reads were mapped to the reference genome sequence and only reads with an identical match or one mismatch were further examined and annotated. The HISAT2 software (https://daehwankimlab.github.io/hisat2/) was used to align the clean reads accurately to the *Citrus maxima* reference genome. Finally, the StringTie (https://ccb.jhu.edu/software/stringtie/) Reference Annotation-Based Transcript assembly method was used to construct and identify both known and novel transcripts from the HISAT2 alignment results.

Gene function was annotated using the following databases: Nr (NCBI non-redundant protein sequences) (https://ccb.jhu.edu/software/stringtie/); Pfam (Protein family)(http://pfam.xfam.org/); KOG/COG (Clusters of Orthologous Groups of proteins) (http://www.ncbi.nlm.nih.gov/KOG/)/ (http://www.ncbi.nlm.nih.gov/COG/); Swiss-Prot (a manually annotated and reviewed protein sequence database) (http://www.uniprot.org/); KO (KEGG Ortholog database) (http://www.genome.jp/kegg/); GO (Gene Ontology) (http://www.geneontology.org/).

Gene expression levels were quantified using the fragments per kilobase of transcript per million fragments mapped (FPKM) method as follows: FPKM = cDNA Fragments/[Mapped Fragments (in millions) × Transcript Length (in kilobases)].

#### 2.3.4. Differential gene expression analysis.

Based on previous studies, this study used a sample size of 12 and 3 biological replicates to identify differentially expressed genes between samples [3,6]. For samples with biological replicates, differential gene expression analysis between two conditions or groups was performed using the DESeq2 software (https://bioconductor.org/packages/release/bioc/html/DESeq2.html). DESeq2 uses negative binomial distribution to model count data and is used to determine differentially expressed genes using RNA sequencing data. P-values were adjusted using the Benjamini and Hochberg’s method to control the false discovery rate. Finally, genes with an adjusted P-value < 0.01 and Fold Change ≥ 1.5 were considered as differentially expressed.

### 2.4. Correlation analysis of the transcriptome and metabolome datasets

Integrated analysis of the DEGs and differentially accumulated metabolites (DAMs) was performed to ascertain the level of pathway enrichment using a correlation heat map, a correlation matrix, and an association network diagram. Subsequently, GO and KEGG pathway analysis was performed to identify enriched pathways that were shared by the DEGs and DAMs.

## 3. Results and analysis

### 3.1. Metabolomic analysis

#### 3.1.1. Quality assessment of metabolomic data.

**Fig 1:**
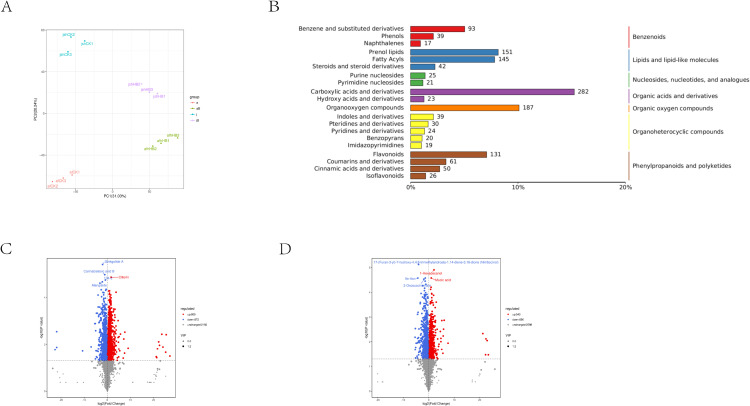
Jinggang honey pomelo metabolome changes in response to Phyllosticta citricarpa. (A) PCA score plot. (B) Select the top 20 Metabolite classification summary based on the HMDB database. (C-D) Volcano plots of differentially expressed metabolites between the afCK _vs_afHB and jsCK_vs_jsHB groups (p-value < 0.05 and Fold Change > 1).

Principal component analysis (PCA) was used to assess the metabolic differences and intra-group variability between the groups. PCA results demonstrated significant differences between groups. This analytical approach was applied to both the sample and control groups for the Jinlan pomelo (designated as ‘af’) and Jinsha pomelo (designated as ‘js’) varieties to evaluate the variation levels within and between the yellow spot disease-affected samples of the two cultivars. PCA results demonstrated distinct clustering of the three biological replicates within each of the four groups. This suggested minimal within-group variation and good replicability, thereby enhancing the reliability of identifying differentially expressed metabolites between the groups. In both cultivars, the PCA scores for the control (CK) and yellow spot disease (HB) groups were significantly different. This indicated significant metabolic differences between the CK and HB groups across both varieties (Fig 1A).

#### 3.1.2. Metabolite analysis.

Metabolite profiling of the ‘af’ and ‘js’ varieties resulted in the identification of 1,425 distinct metabolites across the top 20 distinct categories. This included 93 benzene and its substituted derivatives, 39 phenols, 17 naphthalenes, 151 prenol lipids, 145 fatty acyls, 42 steroids and their derivatives, 25 purine nucleosides, 21 pyridine nucleosides, 282 carboxylic acids and their derivatives, 23 hydroxy acids and their derivatives, 187 organooxygen compounds, 39 indoles and their derivatives, 30 pteridines and their derivatives, 24 pyridines, 20 benzopyrans, 19 imidazopyrimidines, 131 flavonoids, 61 coumarins and their derivatives, 50 cinnamic acids and their derivatives, and 26 isoflavonoids (Fig 1B).

#### 3.1.3. In-depth analysis of differential accumulated metabolites.

**Fig 2:**
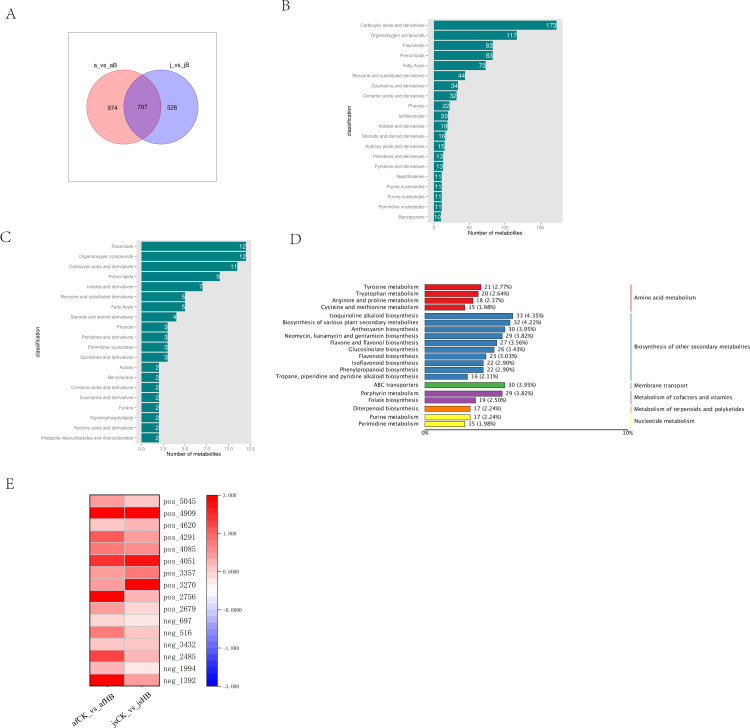
Analysis of the metabolome changes in Jinggang honey pomelo in response to infection by Phyllosticta citricarpa. (A) The Venn diagram shows common DAMs between the afCK_vs_afHB and jsCK_vs_jsHB groups. (B) Classification diagram shows common DAMs in the afCK_vs_afHB and jsCK_vs_jsHB groups. (C) Classification diagram shows DAMs that are co-upregulated in the afCK_vs_afHB and jsCK_vs_jsHB groups. (D) KEGG pathway enrichment map for the common DAMs. (E) Heat map of the 16 flavonoid DAMs.

Volcano plots were used to assess the overall trend of metabolite accumulation and statistical significance of the differences in metabolite content between the two varieties. Variable Importance in Projection (VIP) ≥1 and a P-value <0.05 were used as threshold criteria for screening differential accumulated metabolites (DAMs). We identified 1,681 DAMs in the ‘afCK_vs_afHB’ variety, including 809 up-regulated and 872 down-regulated metabolites (Fig 1C). In the ‘jsCK_vs_jsHB’ group, we identified 1,233 DAMs, including 543 up-regulated and 690 down-regulated metabolites (Fig 1D). Furthermore, 707 DAMs were common among the ‘afCK_vs_afHB’ and ‘ jsCK_vs_jsHB’ varieties (Fig 2A). Predominantly, the DAMs were categorized into carboxylic acids and derivatives, organooxygen compounds, flavonoids, prenol lipids, fatty acyls, coumarins and derivatives, and benzene and its substituted derivatives. A high number of up-regulated DAMs were flavonoids (Fig 2B, C). This suggested a significant association between flavonoids and resistance against yellow spot disease in Jinggang honey pomelo.

#### 3.1.4. Functional enrichment analysis of DAMs.

To elucidate the underlying mechanisms of DAMs in the metabolic pathways, KEGG pathway enrichment analysis was performed for the 707 DAMs identified in the afCK_vs_afHB and jsCK_vs_jsHB groups. The DAMs were enriched in KEGG pathways associated with isoquinoline alkaloid biosynthesis, biosynthesis of various plant secondary metabolites, anthocyanin biosynthesis, ABC transporters, neomycin, kanamycin, and gentamicin biosynthesis, porphyrin metabolism and flavone and flavonol biosynthesis (Fig 2D).

#### 3.1.5. Analysis of important DAMs.

Flavonoids are a class of ubiquitous polyphenolic secondary metabolites in plants. They are classified into various subclasses, including dihydroflavonols, flavonols, and anthocyanidins, and play a crucial role in the plant defense mechanisms against adverse environmental stresses. Flavonoids also exhibit antioxidant activity and anti-inflammatory properties [7]. Sixteen DAMs that were upregulated in both the ‘afCK_vs_afHB’ and ‘ jsCK_vs_jsHB’ varieties were enriched in KEGG pathways associated with the synthesis of flavonoid compounds (Table 1). Among these, kaempferol 3-O-rhamnoside-7-O-glucoside was upregulated by 7.45-fold in the ‘afCK_vs_afHB ‘ group; delphinidin-3-(p-coumaroyl)-rutinoside-5-glucoside was upregulated by 4.81-fold in the ‘ jsCK_vs_jsHB’ variety; quercetin 3-O-beta-D-glucosyl-(1- > 2)-beta-D-glucoside was upregulated by 3.20-fold in the ‘afCK_vs_afHB’ variety. Heatmap analysis of these differential metabolites demonstrated a more pronounced upregulation in the ‘afCK_vs_afHB’group (Fig 2E).

**Table 1:** 16 Differential Metabolites in the KEGG Flavonoid Synthesis Pathway.

ID	Metabolite	Class	a_vs_aB Fold_change	a_vs_aB log2FC	a_vs_aB P value	j_vs_jB Fold_change	j_vs_jB_ log2FC	j_vs_jB P value
pos_2756	Kaempferol3-O-rhamnoside- 7-O-glucoside	Flavonols	174.5086	7.4472	0.0238	1.7594	0.8151	0.0452
neg_1392	Quercetin 3-O-beta-D-glucosyl-(1- > 2)- beta-D-glucoside	Flavonoids	9.2019	3.2019	0.0057	2.2107	1.1445	0.0236
pos_4909	Cyanidin-5-O-beta-D-glucoside3-O-beta-D-sambubioside	Flavonoids	7.2343	2.8548	0.0250	7.8351	2.9700	0.0002
pos_4051	Peonidin-3-(p-coumaroyl)-rutinoside-5-glucoside	Anthocyanidins	4.9560	2.3092	0.0161	6.6521	2.7338	0.0304
neg_2485	Daidzein	Isoflavonoids	4.0790	2.0282	0.0051	1.8796	0.9104	0.0020
pos_4291	Petunidin 3-O-glucoside	Flavonoids	3.5496	1.8277	0.0053	2.0022	1.0016	0.0007
pos_4085	Pelargonidin 3-O-rutinoside 5-O-beta-D-glucoside	Anthocyanidins	2.9812	1.5759	0.0035	2.3464	1.2305	0.0074
neg_516	(-)-Maackiain-3-O-glucosyl- 6’‘-O-malonate	Flavonoids	2.7865	1.4784	0.0008	1.6941	0.7605	0.0157
pos_5045	Malonylgenistin	Isoflavonoids	2.2119	1.1453	0.0361	1.5951	0.6737	0.0047
pos_3357	Delphinidin3-O-beta-D-glucoside5-O-(6-coumaroyl-beta-D-glucoside)	Anthocyanidins	2.1723	1.1192	0.0111	2.7245	1.4460	0.0296
pos_3270	Delphinidin-3-(p-coumaroyl)-rutinoside-5-glucoside	Anthocyanidins	2.1222	1.0856	0.0244	27.9796	4.8063	0.0072
pos_2679	Scolymoside	Flavonoids	2.0362	1.0259	0.0036	1.3490	0.4319	0.0299
neg_1994	Cyanidin3-O-beta- D-sambubioside	Flavonoids	1.9815	0.9866	0.0006	1.2961	0.3742	0.0089
pos_4620	Kaempferol3-sophorotrioside	Flavonols	1.6728	0.7423	0.0075	1.8600	0.8953	0.0091
neg_3432	Isoswertisin 2’‘-rhamnoside	Flavonoids	1.5834	0.6630	0.0010	1.5529	0.6350	0.0138
neg_697	(+)-Sophorol	Flavonoids	1.4257	0.5117	0.0005	1.2934	0.3712	0.0411

### 3.2. Transcriptomic analysis

#### 3.2.1. Screening of differentially expressing genes.

**Fig 3:**
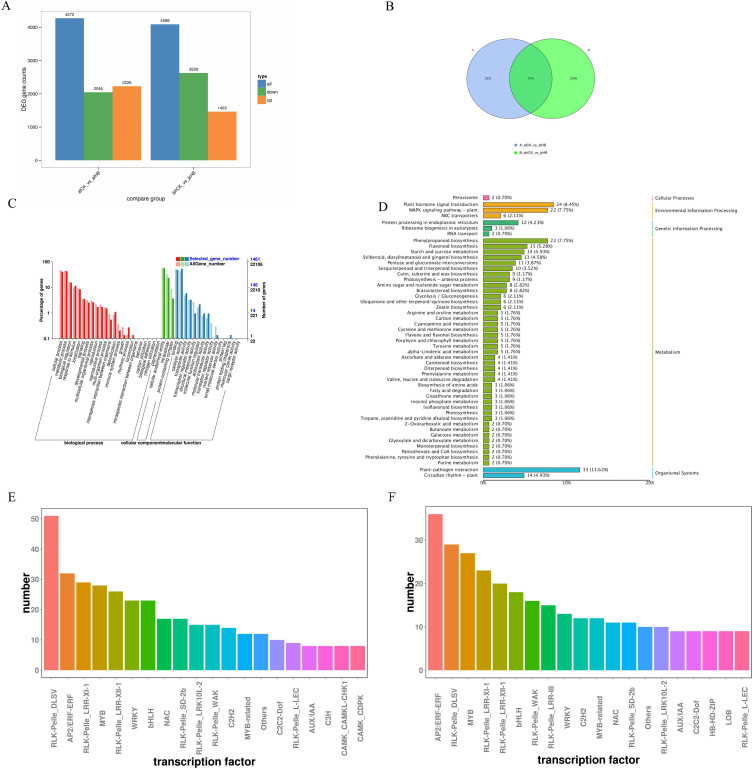
Analysis of the transcriptome changes in the Jinggang honey pomelo varieties in response to infection by Phyllosticta citricarpa. (A) The number of DEGs in the afCK_vs_afHB and jsCK_vs_jsHB groups. (B) Venn diagram shows the number of common DEGs between afCK_vs_afHB and jsCK_vs_jsHB groups. (C) GO analysis of the 1741 common DEGs. (D) KEGG pathway analysis of the 1741 common DEGs. (E-G) Classification diagrams of transcription factors associated with enriched DEGs in the afCK_vs_afHB and jsCK_vs_jsHB groups.

To investigate the disease resistance mechanisms in the ‘afCK_vs_afHB’ and ‘jsCK_vs_jsHB’ varieties of Jinggang honey pomelo, differentially expressed genes (DEGs) were screened using fold change (FC) ≥ 1.5 and FDR (False Discovery Rate) < 0.01 as threshold parameters. In the ‘afCK_vs_afHB’ variety, 4272 DEGs were identified, including 2,226 upregulated and 2,046 downregulated DEGs; in the ‘jsCK_vs_jsHB’ variety, 4,089 DEGs were identified, including 1,463 upregulated and 2,626 downregulated DEGs (Fig 3A).

#### 3.2.2. Gene ontology classification and KEGG pathway enrichment of DEGs.

Venn diagram demonstrated 1,714 shared DEGs between the ‘afCK_vs_afHB’ and ‘jsCK_vs_jsHB’ varieties (Fig 3B). To gain a deeper understanding of the functions of these common DEGs in both ‘afCK_vs_afHB’ and ‘jsCK_vs_jsHB’ varieties, we performed gene ontology (GO) analyses in three main categories, namely biological processes (BP), cellular components (CC), and molecular functions (MF). The top GO-BP terms enriched with the common DEGs were cellular processes, metabolic processes and biological regulation; top GO-CC terms enriched with the common DEGs were cellular anatomical entities, intracellular components, and protein-containing complexes; top GO-MF terms enriched with the common DEGs were binding, catalytic activity, and transporter activity (Fig 3C). The top KEGG pathways enriched with the common DEGs were plant hormone signal transduction, plant-pathogen interaction, phenylpropanoid biosynthesis, MAPK signaling pathway in plants, and flavonoid biosynthesis (Fig 3D).

#### 3.2.3. Identification and analysis of transcription factors.

Transcription factors modulate gene expression and significantly influence biological processes by binding to the regulatory regions of functional genes and altering their expression levels [8]. We identified genes encoding transcription factors within the differentially expressed genes (DEGs) of the ‘afCK_vs_afHB’ and ‘jsCK_vs_jsHB’ varieties. In the ‘afCK_vs_afHB’ variety, we identified 365 transcription factor-related genes belonging to 20 families. The highest number of transcription factor DEGs were found in the *RLK-Pelle* family followed by the *AP2/ERF* and *MYB* families. In the ‘ jsCK_vs_jsHB’ variety, 308 transcription factor-related DEGs were identified belonging to 20 families. The highest number of transcription factor DEGs in the ‘jsCK_vs_jsHB’ variety belonged to the *AP2/ERF-ERF* family followed by the *RLK-Pelle* and *MYB* families. In the ‘afCK_vs_afHB’ variety, majority of DEGs in the *RLK-Pelle*, *AP2/ERF*, and *MYB* families were upregulated, whereas in the ‘jsCK_vs_jsHB’ variety, majority of DEGs in the *RLK-Pelle*, *AP2/ERF*, and *MYB* families were predominantly downregulated (Fig 3E, F).

### 3.3. Integrated metabolome and transcriptome analysis

Integrated analysis of the transcriptome and metabolome data from the ‘afCK_vs_afHB’ and ‘ jsCK_vs_jsHB’ varieties of Jinggang honey pomelo revealed enrichment of common metabolic pathways in both varieties. Specifically, 84 metabolic pathways were associated with the DEGs and DAMs in the ‘afCK_vs_afHB’ variety, and 80 metabolic pathways were associated with the DEGs and DAMs in the ‘ jsCK_vs_jsHB’ variety. Plant hormone signal transduction, phenylpropanoid biosynthesis, and starch and sucrose metabolism were the most significantly enriched pathways in both cultivars.

**Fig 4:**
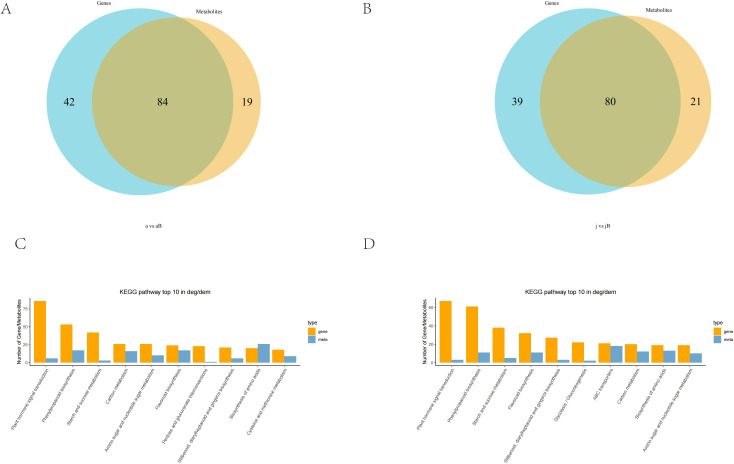
Integrated analysis of the metabolome and transcriptome in Jinggang honey pomelo. (A-B) Venn diagram shows the number of common metabolic pathways represented by the DEGs and DAMs between the afCK_vs_afHB and jsCK_vs_jsHB groups. (C-D) Bar charts show the top 10 pathways with the most DAMs and DEGs in the afCK_vs_afHB and jsCK_vs_jsHB groups.

Metabolites produced by the phenylpropanoid biosynthesis pathway play significant roles in disease resistance and anti-inflammatory effects. This suggested a plausible association between the resistance mechanism against yellow spot disease in Jinggang honey pomelo and the phenylpropanoid biosynthesis pathway metabolites and genes. These findings provide important clues for further investigation into the disease resistance mechanisms of these cultivars and may aid in the development of new resistance strategies. A deeper understanding of the roles of genes and metabolites in these pathways can inform breeding efforts and disease management for Jinggang honey pomelo (Fig 4).

#### 3.3.1. Phenylpropanoid biosynthesis pathway.

**Fig 5:**
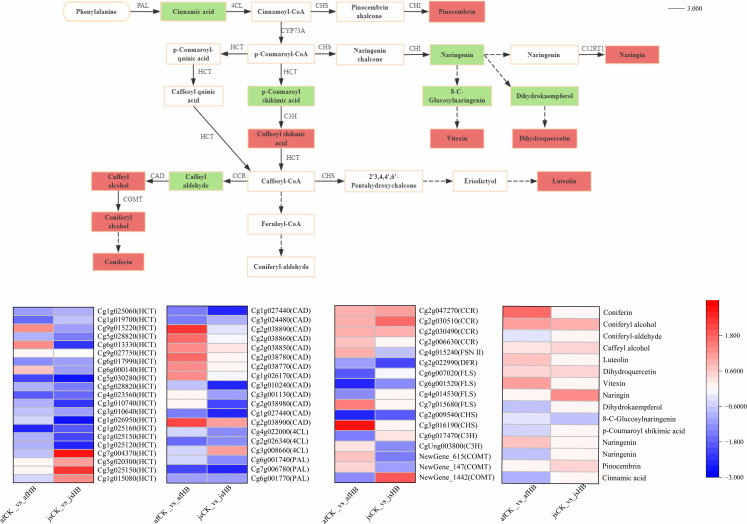
Heat maps of DAMs and DEGs related to the phenylpropanoid biosynthesis and flavonoid biosynthesis pathways. In the metabolic pathways, red represents upregulated expression; green represents downregulated expression. In the heatmaps, colors indicate upregulation or downregulation of DAMs and DEGs; red represents upregulation and blue represents downregulation; darker color indicates higher expression level.

Phenylpropanoid biosynthesis pathway is a major metabolic pathway in plants for the synthesis of several important secondary metabolites, including flavonoids, anthocyanin, and lignin [9]. These secondary metabolites play a significant role in plant growth, development, and stress response [10]. Previous studies have demonstrated that phenylalanine ammonia-lyase (PAL), cinnamate 4-hydroxylase (C4H), and 4-coumarate--CoA ligase (4CL) are key enzymes in the phenylpropanoid biosynthesis and flavonoid biosynthesis pathways [9]. This study demonstrated that phenylpropanoid metabolic pathway was enriched in the yellow spot disease-affected cultivars of Jinggang honey pomelo. The enzymes in the phenylpropanoid metabolic pathway, including PAL, 4CL, hydroxycinnamoyl transferase (HCT), cinnamoyl-CoA reductase (CCR), and cinnamyl-alcohol dehydrogenase (CAD) regulate metabolic reactions that produce a plethora of secondary metabolites, which confer resistance to various external stresses (Fig 5).

#### 3.3.2. Flavonoid biosynthesis pathway.

Flavonoid biosynthesis pathway is a significant branch of the phenylpropanoid biosynthesis pathway, which generates several flavonoid metabolites that accumulate in plants when subjected to stress [11]. Flavonoids are secondary metabolites that protect against various external stresses during plant growth and are active constituents in numerous medicinal plants [12]. They also exhibit a range of biological activities. For example, flavonoids and other polyphenols possess antioxidant properties, enhance insulin secretion, and reduce blood pressure [13]. Previous studies have shown that PAL, 4CL, F3H, CHS, CHI, and FLS are key enzymes in flavonoid biosynthesis [14,15].In this study, HCT, CHS, flavonol synthase (FLS), flavanone 4-reductase (DFR), 5-O-(4-coumaroyl)-D-quinate 3-monooxygenase (C3H) and flavone synthase II (FNS II) were identified as key enzymes in the flavonoid synthesis pathway. Genes encoding CHS*,* FLS, and FNS II were designated as *Cg3g016190,Cg7g015680*, and *Cg4g015240*, respectively, and were upregulated in the ‘afCK_vs_afHB’ variety. Majority of genes encoding CAD and HCT were also upregulated. In contrast, *Cg6g001740* and *Cg4g022000* encoding PAL and 4CL*,* respectively, were downregulated in both the ‘afCK_vs_afHB’ and ‘ jsCK_vs_jsHB’ varieties. Based on the gene heatmap, these DEGs encoding key enzymes exhibited significant upregulation in the ‘afCK_vs_afHB’ variety (Fig 5).

### 3.4. Correlation analysis of the network interaction between DAMs and DEGs

Spearman correlation was used to analyze the network interaction between genes and metabolites. In contrast to Pearson’s correlation analysis, Spearman correlation is used to analyze two sets of data without a clearcut linear relationship [16,17]. Spearman correlation analysis between DAMs and DEGs was performed using the Prism 8.0 (GraphPad, USA) software. We used correlation coefficient |r| ≥ 0.8 and P < 0.05 as threshold parameters to identify DAMs and DEGs with statistically significant relationships.

**Fig 6:**
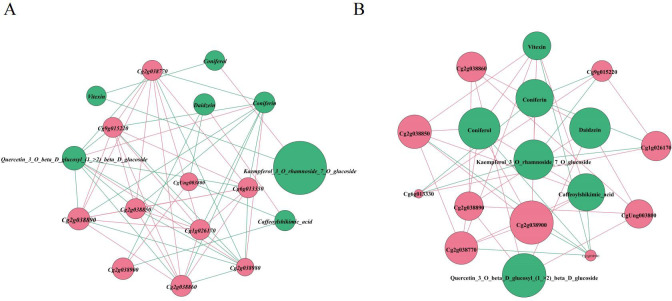
Spearman correlation network diagram. (A-B) Correlation between DAMs and DEGs in the afCK_vs_afHB and jsCK_vs_jsHB groups.

We performed correlation analysis of important and significantly altered DAMs such as daidzein, kaempferol-3-O-rhamnoside-7-O-glucoside, and quercetin-3-O-beta-D-glucosyl-(1- > 2)-beta-D-glucoside, as well as important DEGs and DAMs in the phenylpropanoid biosynthesis pathway (Ko00940) and flavonoid biosynthesis pathway (Ko00941). We identified 10 DEGs and 4 DAMs with significant correlations. Vitexin was the only DAM downregulated in the ‘ jsCK_vs_jsHB’ variety. The remaining metabolites accumulated significantly in both the ‘afCK_vs_afHB’and ‘jsCK_vs_jsHB’ varieties. Vitexin was positively regulated in the ‘jsCK_vs_jsHB’ variety by three genes, *Cg2g038900*, *Cg2g038850*, and *Cg2g038770*, and negatively regulated by the *Cg2g038980* gene*.Cg2g038900*, *Cg2g038850*, and *Cg2g038770* genes encoding CAD were upregulated in both ‘afCK_vs_afHB’ and ‘jsCK_vs_jsHB’ varieties. CAD is a key enzyme in the lignin synthesis pathway and is responsible for converting cinnamyl alcohol into corresponding lignin monomers. This leads to accumulation of lignin monomers, thereby enhancing the strength and defense capacities of the plant cell wall. Daidzein was negatively regulated in ‘afCK_vs_afHB’ by two genes, *Cg2g038900* and *CgUng003800*, but positively regulated in ‘ jsCK_vs_jsHB’ by three genes, *Cg2g038860*, *Cg2g038890*, and *CgUng003800*. Quercetin-3-O-beta-D-glucosyl-(1- > 2)-beta-D-glucoside in ‘afCK_vs_afHB’ was negatively regulated by seven genes, *Cg6g013330*, *Cg9g015220*, *Cg1g026170*, *Cg2g038770*, *Cg2g038850*, *Cg2g038860*, and *Cg2g038890*, and positively regulated by the *Cg2g038980* gene. Quercetin-3-O-beta-D-glucosyl-(1- > 2)-beta-D-glucoside in ‘ jsCK_vs_jsHB’ was positively regulated by three genes, *Cg2g038860*, *Cg2g038890*, and *CgUng003800*. Kaempferol-3-O-rhamnoside-7-O-glucoside in ‘ jsCK_vs_jsHB’ was negatively regulated by three genes, *Cg6g013330*, *Cg9g015220*, and *Cg1g026170* (Fig 6).

## 4. Discussion

### 4.1. Analysis of key DAMs

Based on the threshold criteria of VIP ≥ 1 and P-value<0.05, we identified 1,681 DAMs (809 upregulated and 872 downregulated) in the ‘afCK_vs_afHB’ variety and 1,233 DAMs (543 upregulated and 690 downregulated) in the ‘jsCK_vs_jsHB’ variety. Furthermore, 707 common DAMs were identified in both the varieties. Among these, we observed significant upregulation od several flavonoid compounds. This suggested that resistance against yellow spot disease in Jinggang honey pomelo was closely associated with flavonoids. Kaempferol 3-O-rhamnoside-7-O-glucoside was upregulated by 7.45-fold, followed by delphinidin-3-(p-coumaroyl)-rutinoside-5-glucoside and quercetin 3-O-beta-D-glucosyl-(1- > 2)-beta-D-glucoside. In the Arabidopsis shoots, kaempferol 3-O-rhamnoside-7-O-glucoside, a specific flavonol glycoside, functions as an endogenous inhibitor of polar auxin transport [18]. Flavonols are found in various plant tissues and developmental stages. They participate in plant growth and development, and modulate plant tolerance against biotic and abiotic stress [19]. Delphinidin −3- (p-coumaroyl)-rutinoside-5-glucoside is an anthocyanin and quercetin 3-O-beta-D-glucosyl-(1- > 2)-beta-D-glucoside is a flavonoid. Anthocyanins and flavonoids are associated with anti-inflammatory and antioxidant effects and play a significant role in plant defense mechanisms against various environmental stresses [20].

Integrated analysis of important DAMs in the phenylpropanoid biosynthesis pathway and flavonoid biosynthesis pathway demonstrated that vitexin, daidzein, caffeoyl shikimic acid, coniferyl alcohol, coniferyl aldehyde, coniferin, and dihydroquercetin were important flavonoid compounds that were differentially upregulated. Vitexin is a flavone glycoside belonging to the apigenin class and is found in various foods and medicinal plants [21]. It exhibits a variety of pharmacological properties, including anti-myocardial infarction, anti-inflammatory, analgesic, antioxidant, and anti-tumor effects [22]. Infection by the pathogen causing yellow spot disease can result in vitexin activating the immune system in the Jinggang honey pomelos and inducing systemic acquired resistance (SAR). Vitexin also regulates the levels of plant hormones (salicylic acid and jasmonic acid) and promotes the synthesis of phytoalexins (phenolic and flavonoids) and pathogenesis-related proteins (PR proteins), which have direct inhibitory effects on the pathogens. Vitexin also maintains intracellular redox balance by scavenging reactive oxygen species (ROS), thereby enhancing the survival of Jinggang honey pomelo when infected by Phyllosticta citricarpa [23].

Daidzein is a primary component of soy isoflavones and possesses a broad range of biological activities, including antioxidant and cell cycle regulatory properties [24]. Daidzein inhibits activation of inflammation-related signaling pathways, thereby alleviating the inflammatory response of Jinggang honey pomelo when invaded by the yellow spot pathogen [25]. Caffeoylshikimic acid demonstrates inhibitory effects on a variety of pathogenic bacteria. For example, caffeoylshikimic acid inhibits the growth and reproduction of pathogenic bacteria, thereby enhancing the disease resistance of plants. Furthermore, caffeoylshikimic acid and its derivatives accumulate in the infected plants, thereby directly inhibiting the pathogens. Caffeoylshikimic acid also enhances the mechanical strength of the cell wall by participating in the lignification process of the plant cell wall, thereby enhancing the resistance of Jinggang honey pomelo against the yellow spot pathogen [26,27].

Coniferyl alcohol is an intermediate of lignin biosynthesis with significant antifungal activity and inhibits the growth of several plant pathogenic fungi [28]. Lignin acts as a physical barrier in the plant cell wall and limits the spread of pathogens [29]. Coniferyl alcohol enhances cell wall integrity by participating in lignin synthesis, thereby improving plant defense against pathogens. When the cell wall integrity (CWI) is damaged, plants release damage-associated molecular patterns (DAMPs), which trigger an immune response [30]. Lignin plays a key role in this process. The enzymes involved in lignin synthesis such as p-coumarate 3 – hydroxylase (C3H) and cinnamoyl-CoA reductase (CCR) are also associated with the plant disease resistance mechanism [30]. Coniferyl aldehyde protects plant cells from oxidative damage caused by pathogen invasion by scavenging free radicals and inhibiting oxidative stress. Coniferyl aldehyde is an important intermediate in lignin synthesis and enhances the mechanical strength of plant cell walls through the lignification process. The accumulation of coniferyl aldehyde in infected Jinggang honey pomelos directly inhibits the invading pathogens [31,32].

Coniferin is a soluble phenolic compound participating in the lignification process of plant cell walls. Lignin deposition is an important plant defense mechanism against pathogen invasion because it enhances mechanical strength of the cell wall, thereby restricting the penetration and spread of the yellow spot pathogen in Jinggang honey pomelo [33]. Dihydroquercetin is a plant-derived dihydroflavonol with strong antioxidant activity through which it protects plant cells from oxidative damage caused by pathogen invasion and maintains intracellular redox balance. Dihydroquercetin also promotes synthesis of other compounds with plant defense functions. For example, it promotes synthesis of flavonoids such as myricetin, (+)-gallocatechin, and (+)-catechin. These compounds are associated with antioxidant, antiviral, and antibacterial activities, thereby reducing the damage of Jinggang honey pomelo to *Alternaria alternata* [34,35].

In response to external stress environments, plants increase cell wall strength by altering the cell wall composition [36]. Cell wall strength plays a significant role in plant growth, development, and stress resistance. Dihydroquercetin protects structural integrity of plant cells, especially in response to cell damage caused by pathogen invasion. During infection by the yellow spot pathogen, dihydroquercetin enhances disease resistance by reducing cell apoptosis and necrosis and maintaining the normal function of cells [34]. Dihydroquercetin, a dihydroflavonol, is a precursor of anthocyanins, a type of phenylpropanoids. In recent years, antioxidant, anti-apoptotic, antiviral, and anti-tumor activities of dihydroquercetin have been discovered [37,38]. Most flavonoid metabolites possess anti-inflammatory and antioxidant properties and accumulate in significant amounts in the ‘afCK_vs_afHB’ and ‘ jsCK_vs_jsHB’ varieties after disease onset. This suggests that flavonoids play a crucial role in the resistance against yellow spot disease in the Jinggangshan honey pomelos.

### 4.2. Analysis of important DEGs

Transcriptome analysis with FC ≥ 1.5 and FDR < 0.01 as threshold parameters resulted in the identification of 4,272 DEGs (2,226 upregulated and 2,046 downregulated) in the ‘afCK_vs_afHB’ variety and 4,089 DEGs (1,463 upregulated and 2,626 downregulated) in the ‘ jsCK_vs_jsHB’ variety. Our results showed significant enrichment of key enzymes in the phenylpropanoid metabolic pathway, including phenylalanine ammonia-lyase (PAL), 4-Coumarate: CoA ligase (4CL), hydroxycinnamoyl-CoA: shikimate hydroxycinnamoyl transferase (HCT), cinnamyl-alcohol dehydrogenase (CAD), and cinnamoyl-CoA reductase (CCR). We also identified differential expression of enzymes in the flavonoid synthesis pathway, including HCT, chalcone synthase (CHS), flavonone synthase (FLS), dihydroflavonol 4-reductase (DFR), flavone synthase II (*FNS II*), and p-Coumarate 3-hydroxylase (C3H). HCT is a pivotal enzyme regulating lignin metabolism, whereas CHS is a key enzyme in the flavonoid metabolic pathway [19]. Both HCT and CHS regulate the synthesis of flavonoid substances. HCT silencing or CHS overexpression enhances flavonoid synthesis and is implicated in plant resistance to pests and diseases [39,40]. The *Cg5g020300* gene encoding HCT is upregulated in both the afCK_vs_afHB and jsCK_vs_jsHB varieties. Furthermore, *Cg6g013330*, *Cg9g015220*, *Cg7g004370*, *Cg1g015080* genes are also upregulated in the afCK_vs_afHB and jsCK_vs_jsHB varieties. The upregulation of the genes encoding HCT promotes synthesis of coumarin compounds, which have antibacterial and antioxidant properties that directly inhibit the growth of pathogenic bacteria and scavenge free radicals in plant cells. Coumarin compounds also combine with cell wall components to enhance the strength and stability of the cell wall, thereby enhancing the resistance against pathogenic bacteria and reducing damage to the Jinggang honey pomelo [41,42].The CHS-encoding gene *Cg3g016190* was upregulated in the ‘afCK_vs_afHB’ variety by a log_2_FC value of 2.69. CHS is the rate-limiting enzyme in the flavonoid synthesis pathway. Its upregulation increases the synthesis of flavonoids and isoflavonoids, which are antibacterial compounds in plants. They directly inhibit the growth of the pathogen causing citrus yellow spot, thereby promoting the defense and disease resistance of Jinggang honey pomelo [43]. The FLS-encoding gene *Cg7g015680* gene was also upregulated in the ‘afCK_vs_afHB’ variety by a log_2_FC value of 1.41. FLS is a key enzyme in the flavonoid synthesis pathway. Its upregulation promotes the synthesis of flavonol compounds such as quercetin and kaempferol. Flavonoids have antibacterial and antioxidant properties. Flavonol compounds enhance the strength of the cell wall by binding to cellulose and lignin, thereby enhancing resistance of plants against pathogens. Therefore, its upregulation in the infected Jinggang pomelo can inhibit the growth and reproduction of the pathogen [44,45]. The FNS II-encoding gene *Cg4g015240* was upregulated with an log_2_FC value of 0.90 in the ‘afCK_vs_afHB’ variety. The upregulation of FNS II promotes the synthesis of flavonoids such as flavones and flavonols, which have antioxidant properties and scavenge reactive oxygen species in the plant cells. They reduce the damage to the plant cells caused by the yellow spot pathogen in Jinggang honey pomelo [41].

Enzymes such as PAL, C4H,4CL, COMT, CCR, and CAD are key enzymes in lignin synthesis [46]. In this study, genes encoding COMT, CAD, and CCR enzymes were upregulated. For example, CAD-encoding genes *Cg2g038900* and *Cg2g038890* were upregulated in the afCK_vs_afHB variety with log_2_FC values of 2.25 and 2.29, respectively. COMT-encoding genes *NewGene_147* and *NewGene_615* were upregulated in the afCK_vs_afHB variety with log_2_FC values of 0.53 and 0.72, respectively. CCR-encoding genes were upregulated in both the afCK_vs_afHB and jsCK_vs_jsHB varieties. The upregulation of COMT in Jinggang honey pomelo promotes synthesis of lignin monomers and increases lignin content. Lignin deposition enhances cell wall strength and rigidity and provides a physical barrier against the invasion and spread of the yellow spot disease-causing pathogen in Jinggang honey pomelo [47]. CAD-encoding genes such as *Cg2g038900*, *Cg2g038890*, *Cg1g026170*, and *Cg2g038770* are upregulated in the Jinggang honey pomelo. CAD is a key enzyme in the lignin synthesis pathway and is responsible for converting cinnamyl alcohol into lignin monomers. Therefore, CAD significantly promotes lignin synthesis and enhances the plant cell wall strength [48,49]. CCR-encoding genes such as *Cg2g047270*, *Cg2g030490*, *Cg2g030510*, and *Cg2g047270* are upregulated in both varieties of pomelo. CCR is the first key enzyme in the lignin biosynthesis pathway and is responsible for reducing cinnamoyl-CoA into cinnamyl alcohol. Therefore, upregulation of CCR-encoding genes increases the levels of CCR, promotes the synthesis of lignin, and enhances the structural integrity of the plant cell wall [50,51].

Several genes encoding CAD and *HCT* were also upregulated but genes encoding PAL (*Cg6g001740*) and 4CL (*Cg4g022000*) were downregulated in both ‘afCK_vs_afHB’ and ‘ jsCK_vs_jsHB’ varieties. DFR protein is essential for anthocyanin synthesis. Many plants enhance anthocyanin levels under abiotic stress by increasing *DFR* expression levels. Temperature is another critical factor affecting anthocyanin synthesis. Anthocyanin synthesis is increased at low temperatures and inhibited at high temperatures [20]. In this study, DFR-encoding gene *Cg2g022990* was downregulated in both the ‘afCK_vs_afHB’ and ‘ jsCK_vs_jsHB’ varieties, possibly due to high temperatures at the time of sampling. The *4CL* gene is a regulatory gene and its expression is controlled during developmental stages [52]. *PAL* enzyme activity fluctuates during plant growth and development. Furthermore, its activity is subjected to feedback inhibition by end products, as well as inhibitors such as cinnamic acid, p-coumaric acid, and amino acids such as histidine and tryptophan. Furthermore, the inhibitor sensitivity of *PAL* can vary between different plant sources as well as between different plant tissues [53]. In this study, downregulation of genes encoding *PAL* and *4CL* in both ‘afCK_vs_afHB’ and ‘ jsCK_vs_jsHB’ varieties may be associated with the developmental stage of the pomelo and the regulation by end products.

### 4.3. Transcription factor analysis

Transcription factors play a significant role in biological growth and development by regulating gene expression. They also play integral roles in the response to abiotic stresses [54]. In both the ‘afCK_vs_afHB’ and ‘ jsCK_vs_jsHB’ varieties, genes encoding transcription factors such as *RLK-Pelle*, *AP2/ERF*, and *MYB* were differentially expressed. Receptor-like kinases (RLKs) are significantly associated with the regulation of plant growth, development, and defense against diseases [55,56]. For example, in Arabidopsis, 49% (284 out of 577) of the *RLK* genes were differentially expressed under one or more stress conditions, thereby highlighting the significant role of the *RLK* family in stress responses [57]. The *MYB* family of transcription factors facilitate transcription of numerous key enzymes in the flavonoid synthesis pathway, thereby enhancing the synthesis of flavonoid compounds [58]. In the ‘afCK_vs_afHB’ variety, several genes encoding key enzymes in the flavonoid synthesis pathway, including *HCT*, *CAD*, *CHS*, and *FLS*, are upregulated. Furthermore, four genes belonging to the *AP2/ERF* family are differentially expressed. The *AP2/ERF* gene family is implicated in stress responses to abiotic stresses, including intense light, elevated temperatures, and strong light exposure [59]. It is hypothesized that under conditions of high temperature stress, intense light, and pathogen attacks, plants upregulate transcription factors such as *RLK-Pelle*, *AP2/ERF*, and *MYB* to promote biosynthesis of flavonoid compounds to combat external stressors.

## 5. Conclusion

This study comprehensively analyzed the metabolome and transcriptome of Jinggangshan pomelo varieties infected by the yellow spot disease pathogen and identified 1,681 DAMs and 4,272 DEGs in the ‘afCK_vs_afHB’ variety, as well as 1,233 DAMs and 4,089 DEGs in the ‘ jsCK_vs_jsHB’ variety. Flavonoid compounds such as vitexin, daidzein, kaempferol-3-O-rhamnoside-7-O-glucoside, quercetin-3-O-beta-D-glucosyl-(1- > 2)-beta-D-glucoside, caffeoylshikimic acid, coniferol, coniferin, and dihydroquercetin, were all significantly upregulated. Flavonoid metabolites such as vitexin, daidzein, and coniferol are associated with antioxidant, anti-inflammatory, and protective effects against external stresses, thereby suggesting their significant role in the disease resistance mechanism of the Jinggangshan pomelo varieties against yellow spot disease. Key genes encoding enzymes for the synthesis of flavonoid compounds such as HCT (*Cg6g01333*0 and *Cg9g015220*), CAD (*Cg2g038900*, *Cg2g038980*, *Cg1g026170*, *Cg2g038770*, etc.), C3H (*CgUng003800*), *CHS* (*Cg3g016190)*, and FLS (*Cg3g016190* and *Cg7g015680*) were differentially expressed. This suggested transcriptional regulation of the flavonoid pathway enzymes played a key role in the disease resistance mechanism of Jinggangshan pomelo against yellow spot disease. Compared to the ‘ jsCK_vs_jsHB’ variety, the ‘afCK_vs_afHB’ variety showed increased accumulation of flavonoid compounds and higher expression of key genes involved in the synthesis of flavonoid pathway genes. This suggested that disease resistance was more effective in the ‘afCK_vs_afHB’ variety. Furthermore, genes such as *Cg7g015680*, *Cg3g016190*, *Cg2g038890*, *Cg5g020300*, *Cg4g015240*, and *Cg2g006630*, which are involved in the synthesis of disease-resistant compounds such as flavonoids and lignin are upregulated. These genes can serve as candidate biomarkers for the early screening of plants with disease-resistance potential, thereby improving the efficiency of breeding disease-resistant varieties.
